# Alcohol Misuse May Increase the Severity of COVID-19 Infections

**DOI:** 10.1017/dmp.2020.452

**Published:** 2020-11-18

**Authors:** Ebrahim Abbasi-Oshaghi, Fatemeh Mirzaei, Iraj Khodadadi

**Affiliations:** 1 Department of Clinical Biochemistry, School of Medicine, Hamadan University of Medical Sciences, Hamadan, Iran; 2 Department of Anatomy, School of Medicine, Hamadan University of Medical Sciences, Hamadan, Iran; 3 Department of Clinical Biochemistry, Hamadan University of Medical Sciences, Hamadan, Iran

**Keywords:** alcohol, COVID-19, inflammation, lung, pneumonia

Alcohol misuse is recognized as a major health problem worldwide. Chronic alcohol consumption damages nearly all of the body organs and increases the susceptibility of the host to various diseases, such as acute respiratory distress syndrome and pneumonia, the most severe complications of the novel coronavirus disease (COVID-19).^1^ Lung disorders are the main cause of death in COVID-19 patients.^2^ Interestingly, chronic alcohol consumption is accompanied with a twofold to ninefold higher risk of pneumonia.^3^

At present, there are no potential medicines for the prevention of COVID-19.^2^ Some people believe that alcohol consumption is beneficial for the prevention and treatment of COVID-19.^4^ However, alcohol misuse may enhance susceptibility to COVID-19 infection for numerous reasons. Alcohol misuse has detrimental effects on local and systemic immune responses and predisposes the lung to viral or bacterial infections.^5^ Reduced immune system activity may be considered as a main comorbidity in an alcoholic subject, as alcoholic subjects experienced a higher mortality of pneumonia as compared with non-alcoholic subjects. Alcohol consumption significantly upregulated the lung transforming growth factor β-1 (TGF β-1), a cytokine involved in the acute lung injury (ALI).^3^ Long-term alcohol consumption is associated with increased levels of pro-inflammatory markers such as interleukin 6 (IL-6), tumor necrosis factor alpha (TNF-α), and interleukin-1β (IL-1β) levels. Chronic alcohol consumption also is associated with elevated cytotoxic T lymphocytes (CD8^+^ T-cell) proliferation and activation, reduced native T-cells, loss of T-cell numbers, and reduces the alveolar macrophage activity. Interferons are produced in response to a virus infection and currently are administered for the management of COVID-19 infection.^2^ Alcohol consumption significantly decreases interferon secretion and contributes to an elevated risk of viral infections.^5^ Moreover, alcohol reduces macrophage and neutrophil function in the lung, as well as reduces the clearance of pathogens from the respiratory system and facilitates the movement of pathogen into the lung, consequently reducing pathogen killing.^3^ Furthermore, chronic alcohol consumption damages neutrophil recruitment and reduces neutrophil production, and interferes with alveolar macrophage activity.^5^

Alcohol misuse can elevate the risk of aspiration and inhibit the normal cough reflex, and alcoholic individuals often require mechanical ventilation more than non-alcoholic individuals.^3^

Chronic alcohol consumption may raise the risk of severe bacterial and viral infections by altering the lung inflammatory markers (eg, CD8 T-cell). It has been reported that chronic alcohol use enhances the risk of severe pulmonary disease and mortality associated with influenza viral infections, probably by increasing edema and neutrophilia, as well as loss of influenza-specific CD8 T-cell population.^6^

Chronic alcohol consumption exacerbates local and systemic oxidative stress, as well as reduces antioxidant capacity in the alveolar space, leading to ALI and alveolar epithelial dysfunction.^5^ Alcohol misuse leads to a significant decrease in alveolar glutathione levels. Depleted antioxidant capacity in the lung may deactivate α-1 proteinase inhibitor, an event which in turn increases the emphysema risk.^3^ In addition to oxidative stress, alcohol also changes the function of normal alveolar barrier and alveolar type II cells. Chronic alcohol consumption interferes with the formation and function of tight junctions within the pneumocytes. These processes can alter epithelial lining fluid and lead to inflammation, mucus stasis, and increase the risk of lung infections.^3^ Finally, long-term drinking is closely related to malnutrition with zinc and vitamin deficiency, as well as various chronic disorders that may increase the risk of pneumonia and COVID-19 infection.^3^
